# Hippocampal CCR5/RANTES Elevations in a Rodent Model of Post-Traumatic Stress Disorder: Maraviroc (a CCR5 Antagonist) Increases Corticosterone Levels and Enhances Fear Memory Consolidation

**DOI:** 10.3390/biom10020212

**Published:** 2020-02-01

**Authors:** José Joaquín Merino, Vilma Muñetón-Gomez, César Muñetón-Gómez, María Ángeles Pérez-Izquierdo, María Loscertales, Adolfo Toledano Gasca

**Affiliations:** 1 Dpto. Farmacología, Farmacognosia y Botánica, Facultad de Farmacia, Universidad Complutense de Madrid (U.C.M). c/ Plaza Ramón y Cajal s/n, 28040 Madrid, Spain; 2 Universidad de La Salle Center, Facultad de Ciencias Agropecuarias, Av. Carrera 7. # 179-03 (sede norte), Bogotá, Colombia; hvcmg01@hotmail.com (V.M.-G.); cemuneton@unisalle.edu.co (C.M.-G.); 3 Psychobiology Dept. Universidad Nacional de Educación a Distancia, UNED, 28040 Madrid, Spain; maperez@psi.uned.es; 4 Harvard Medical School, MGH, Massachussets General Hospital, 185 Cambridge St, Boston, MA 02114, USA; mloscertales@mgh.harvard.edu; 5 Department of Neuroanatomy, Instituto Cajal (CSIC), c/ Dr. Arce, 28.002 Madrid, Spain; atoledano@cajal.csic.es

**Keywords:** post traumatic stress disorder, CCR5/RANTES chemokines, neural plasticity, chronic stress restraint, fear learning, neuro repair, neuroinmunology, neuroimmunomodulation

## Abstract

*Background*: Contextual fear conditioning (CFC) is a rodent model that induces a high and long-lasting level of conditioning associated with traumatic memory formation; this behavioral paradigm resembles many characteristics of posttraumatic stress disorder (PSTD). Chemokines (chemotactic cytokines) play a known role in neuronal migration and neurodegeneration but their role in cognition is not totally elucidated. *Aim*: We ascertain whether CCR5/RANTES beta chemokines (hippocampus/prefrontal cortex) could play a role in fear memory consolidation (CFC paradigm). We also evaluated whether chronic stress restraint (21 days of restraint, 6-h/day) could regulate levels of these beta chemokines in CFC-trained rats; fear memory retention was determined taking the level of freezing (context and tone) by the animals as an index of fear memory consolidation 24 h after CFC training session; these chemokines (CCR5/RANTES) and IL-6 levels were measured in the hippocampus and prefrontal cortex of chronically stressed rats, 24 h after CFC post-training, and compared with undisturbed CFC-trained rats (Experiment 1). In Experiment 2, rats received 1 mA of footshock during the CFC training session and fear memory consolidation was evaluated at 12 and 24 h after CFC training sessions. We evaluated whether RANTES levels could be differentially regulated at 12 and 24 h after CFC training; in Experiment 3, maraviroc was administered to rats (i.m: 100 mg/Kg, a CCR5 antagonist) before CFC training. These rats were not subjected to chronic stress restraint. We evaluated whether CCR5 blockade before CFC training could increase corticosterone, RANTES, or IL-6 levels and affects fear memory consolidation in the rats 24-h post-testing compared with vehicle CFC-trained rats. *Results*: Elevations of CCR5/RANTES chemokine levels in the hippocampus could have contributed to fear memory consolidation (24 h post-training) and chronic stress restraint did not affect these chemokines in the hippocampus; there were no significant differences in CCR5/RANTES levels between stressed and control rats in the prefrontal cortex (Experiment 1). In Experiment 2, hippocampal CCR5/RANTES levels increased and enhanced fear memory consolidation was observed 12 and 24 h after CFC training sessions with 1 mA of footshock. Increased corticosterone and CCR5/RANTES levels, as well as a higher freezing percentage to the context, were found at 24 h CFC post-testing in maraviroc-treated rats as compared to vehicle-treated animals (experiment-3). Conversely, IL-6 is not affected by maraviroc treatment in CFC training. *Conclusion*. Our findings suggest a role for a hippocampal CCR5/RANTES axis in contextual fear memory consolidation; in fact, RANTES levels increased at 12 and 24 h after CFC training. When CCR5 was blocked by maraviroc before CFC training, RANTES (hippocampus), corticosterone levels, and fear memory consolidation were greater than in vehicle CFC-trained rats 24 h after the CFC session.

## 1. Introduction

Immune responses can affect neural plasticity in brain areas (hippocampus and cortex); acute stress enhances immune responses and chronic stress restraint provokes immunosuppression [1,2,3,4,5,6,7]. Chronic stress restraint provokes behavioral deficits in hippocampal-dependent tasks in rats [8,9,10] and corticosterone, the stress hormone, affects synaptic terminal structure in the hippocampus [11,12] and alters dendritic spine morphology in the rat medial prefrontal cortex [6].

Cognition can be interfered with by blocking immune receptors in the central nervous system [5]. The association of immunomodulatory mechanisms with PTSD-like behavior alteration and immune responses is not well understood. Chemokines (chemotactic cytokines) are G-coupled proinflammatory cytokines involved in neuromodulation, neuronal migration [13,14]. RANTES (regulated on activation, normal T-cell) is a small, secreted 8–10 kD chemokine involved in chemoattraction in eosinophils, monocytes, and certain T leukocyte subsets. The ligand (RANTES: Regulated on Activation, Normal T Cell Expressed and Secreted) binds to CCR5 [15] and also recognizes CCR1 chemokine receptors [16]. The CCR5 antagonist called maraviroc specifically blocks CCR5 while Met-RANTES blocks CCR1 and CCR5 [17]. The CCR5/RANTES axis also contributes to neurodegeneration in the hippocampus [18,19,20] as well as protecting neurons against insults in vitro [21]. Certain chemokines (CXCR4/SDF1 alpha) regulate neuronal excitability [22,23]. These chemokines are detected by molecular techniques in the hippocampus [24,25], which plays a role in contextual fear conditioning (CFC) [26].

Recent evidence supports a role for immune dysfunction in psychiatric conditions such as post traumatic stress disorder (PTSD) [8]. The contextual fear conditioning paradigm (CFC) is a PSTD rodent model that induces a high and long-lasting level of conditioning associated with traumatic memory formation [27,28]. This is a typical Pavlovian conditioning behavior [26]; the neutral stimulus that did not elicit emotional responses is followed by an aversive stimulus (footshocks) in CFC-trained rats using 1 mA footshocks; the re-exposure to the context (neutral stimulus) associates the neutral stimulus with an aversive condition (footshock). These freezing levels correlated with corticosterone levels in CFC-trained rats at 1 mA footshock [28]; the freezing is considered an index of fear memory (context and tone) in this CFC behavioral paradigm; the freezing is evaluated at 12 and 24 h after CFC training as an index of fear memory consolidation [28]. This CFC paradigm reproduces many characteristics of PSTD, such as the persistence of traumatic memory [29]. In fact, PSTD patients re-experience the traumatic event with distressing recollections, flashbacks, and psychologic distress after encountering a stimulus that is reminiscent of the trauma [30,31]. The involvement of the CCR5 chemokine receptor in cognition and fear learning is unconfirmed. We try to resolve this question: are CCR5/RANTES chemokines involved in traumatic memory consolidation in chronically stressed rats?

### Aim

- We studied whether chronic stress restraint could increase CCR5/RANTES chemokine as well as IL-6 levels in the hippocampus/prefrontal cortex (PFC) of rats subjected to 21 days of restraint; we evaluate if these chemokines play a role in fear memory consolidation in this CFC paradigm (1 mA, Experiment 1). These beta chemokines were measured 24 h after CFC training.
- We evaluated whether CCR5/RANTES chemokine levels are differentially regulated at 12 and 24 h post-training in CFC-trained rats with a footshock intensity of 1 mA (Experiment 2).
- Once we confirmed elevated CCR5/RANTES levels at 12 and 24 h after CFC training (1 mA), we evaluated whether maraviroc (a CCR5 blocker, i.m injection: 100 mg/Kg) treatment administered for 3 consecutive days before CFC fear training could affect RANTES/IL-6 and corticosterone levels and also impairs fear memory consolidation 24 h after the CFC session (Experiment 3).

## 2. Material and Methods

### 2.1. Animals

Male *Wistar* rats from Harlan Iberica (Barcelona, Spain), weighing from 165 to 185 g, were caged in groups of three animals with free access to food and water under controlled temperature (22 ± 2 °C) and light conditions (12 h:12 h light-dark cycle, light on at 8 A.M). All animals were handled daily. According to their weight, animals were distributed into several experimental groups, including undisturbed control rats (without stress or non-CFC trained rats).

### 2.2. Behavioral Methods

#### 2.2.1. Chronic Stress Restraint Procedure

All stress sessions took place in a room close to the colonies’ cages in the stabulary room. The daily stress session was done by restraining rats for 21 consecutive days (6-h/day) in plastic restrainers secured to the head and tail end with clip (from 09:00 a.m. to 15:00 p.m.) [27,28].

#### 2.2.2. Contextual Fear Learning Paradigm: A Behavioral Paradigm of PSTD

Rats were trained in a rodent box (30 × 37 × 25 cm) floored with 30 sheets of stainless steel, through which animals received a footshock of 1 mA from a shock generator (LEICA I.C, Model L-I100-26, Spain) connected to the floor during the training session. Briefly, rats were trained for 5 min in the contextual fear conditioning model. During this training session, animals were allowed to explore the environment for 180 s and, after this time, received three consecutive footshocks of 1 mA of intensity every 60 s, following our previous protocols [27,28]. During the training sessions, all undisturbed rats remained in their cages without receiving footshocks or other treatments. Thus, this training session consisted of three footshocks of 1 mA intensity in the conditioning chamber, producing effects that resemble many characteristics of PSTD [28]. Memory formation was evaluated by freezing percentage to the context and tone as an index of fear memory in these CFC-trained rats with 1 mA footshocks; the fear memory consolidation was evaluated at 12 or 24 h after the CFC training session (depending on the experiment); freezing percentage at 24 h CFC post-training is considered as an index of conditioned fear memory consolidation, and it was evaluated for 8 min. However, during this fear memory retention phase evaluated 12/24 h after the CFC session, rats did not receive any footshock [27,28]. This followed behavioral protocol was previously published by us.

Last, CCR5/RANTES protein levels were measured by ELISA/Western blot in crude synaptosomes (hippocampus/cortex), following our own protocols [27,28]. All rats were sacrificed one day after the induction of chronic stress and also 1 day after the end of the CFC training; the hippocampus was dissected (hippocampus and prefrontal cortex) and stored at −80 °C for further biochemical evaluation of chemokines.

### 2.3. Biochemical Methods

#### 2.3.1. Synaptosomes Isolation (Hippocampus and Prefrontal Cortex)

Synaptosomes were obtained following a protocol modified from Lynch and Voss (1991). Briefly, the hippocampus was dissected and homogenized in 1 mL of lysis buffer (10 volumes), containing 0.32 M sucrose, HEPES 5 mM, 1 μg/μL aprotinin, 1 μg/μL leucopeptin, 1 μg/μL peptastine, and 1 mM DTT. These homogenates were centrifuged for 5 min at 1000× *g*, 4 °C using a JA 20.21 rotor. The supernatant was centrifuged for 15 min at 15000× *r.p.m*, 4 °C. After removing this supernatant, the final pellet contains synaptosomes, which were resuspended in PBS 1 X buffer plus HEPES 5 mM, 1 μg/μL aprotinin, 1 μg/μL leucopeptin, 1 μg/μL peptastine, and 1 mM DTT. The total protein estimation was quantified by Bradford; finally, absorbance was read at 595 nm in a DIGYSCAN spectrophotometer (UNED, Madrid).

#### 2.3.2. ELISA CCR5/RANTES Protein Levels (Crude Synaptosomes)

The CCR5/RANTES delta chemokines were measured by ELISA in crude synaptosomes following the manufacturer’s instructions and own protocols [28]. The used kits for ELISA were RANTES (#MMR00 R&D system, MN, USA); CCR5 (#ABIN626788 antibodies-online company, Germany, detection range: 0.312 ng/mL—20 ng/mL), IL-6 kit (# R6000B, R&D, MN, USA). The detection limit for RANTES is 2 pg/mL. Briefly, 10 mg of samples were loaded in their respective plate in order to detect these markers; the capture antibody (concentration 10 μg/μL) was incubated overnight (RANTES: o/n) at 4 °C in PBS (phosphate buffer saline). After four washes with PBS plus 0.05% Tween 20, plates were incubated with a biotinylated secondary antibody (1:500 dilution) for 2 h at room temperature (RT). After 3 washes, the signal was enhanced by adding streptavidin-HRP (1:5000, R&D System, MN, USA) to the plate (30 min). The reaction was allowed to develop in dark conditions by adding OPD 1 μg/μL (ortophenylenediamine, Sigma, Madrid, Spain) in citrate buffer for 10 min and 30% H_2_O_2_. After stopping the reaction with H_2_SO_4_, the absorbance was read at 492 nm wavelength (DIGYSCAN spectrophotometer reader) following our own protocol [28].

#### 2.3.3. Western Blot for CCR5 Detection in Crude Synaptosomes

Briefly, CCR5 was quantified in synaptosomes from the hippocampus by Westerm blot. Samples were homogenized in 500 μL of buffer (PBS/0.1% Nonidet P-4/0.1% SDS/0.5% deoxycholic acid) containing aprotinine (5.7 μg/mL), sodium vanadate (1 mmol/l); and phenylmethylsulfonyl fluoride (100 μg/μL); all these reagents were added immediately after the homogenization procedure. After centrifugation (15000 × *r.p.m*, 20 min), aliquotes from the supernatants were collected and stored at −20 °C until further biochemical analysis.

Synaptosomes from the hippocampus were boiled for 3 min at 90 °C in 30 mM Tris-HCl buffer at pH 7.4, containing 0.05% lauryl dodecyl sulphate sodium (SDS) and beta-mercaptoethanol. Equal amounts of proteins were loaded (40 μg of total protein) and transferred to PVDF membranes at 1 mA/cm^2^ (1A, 200 v during 1 h in a TE 22 Transfer system, Amersham Pharmacy, Madrid, Spain). After blocking nonspecific binding, samples were overnight incubated (o/n) with 5% of milk powered milk free of saturated fat 1:1 in TBS-Tween 20 in 50 mM Tris-HCl, pH = 8, 138 mM NaCl, 0.05% Tween 20 (TBST); the CCR5 antibody was incubated o/n at 12 μg/μL (AbCAM, UK; # ab65850). This CCR5 polyclonal antibody recognizes the N-terminal region of CCR5) and does not cross-react with other chemokine receptors. Beta actine was included as a loading control (stripping) at 1:5000 in TBS-Tween. After blocking membranes with TBS-T in the NAP blocker (*Genotech*), the primary CCR5 antibody was added at 1:500. The secondary anti rabbit HRP horseradish-peroxidase-conjugated antibody was used at 1:20000 for 1 h at room temperature. Finally, blots were developed with the ECL+ chemoluminescence system (*Amersham Pharmacia, Spain*) and densitometry of bands expressed as a percentage of controls.

#### 2.3.4. Corticosterone Levels

Trunk blood was collected by centrifugation and pooled in heparinized vials for centrifugation at 2780 rpm for 10 min at 4 °C. Plasma was collected out and stored at −20 °C until assays were performed. Plasma B was assayed in duplicate with the RIA kit (ICN Biomedical Inc. Costa Mesa, California, USA). This intra-assay coefficient of variation was 4.4%.

## 3. Results

### 3.1. Experiment 1: Effect of Chronic Stress Restraint and Fear Learning in CCR5/RANTES Levels (Hippocampus/Prefrontal Cortex)

In Experiment 1, we evaluate whether chronic stress restraint and/or contextual fear conditioning training (CFC) increase hippocampal/PFC CCR5/RANTES protein levels 24 h post-testing. We quantified freezing levels like an index for memory consolidation at 24 h after the CFC session, following our own protocols [28]. Animals were subjected to 21 days of chronic stress restraint in plastic restrainers. One day later (day 23), a subgroup of animals from the “stress condition” (*n* = 16) or “undisturbed” (*n* = 16) groups were trained in a contextual fear conditioning paradigm (CFC) paradigm with 1 mA footshocks. Rats were re-exposed to the context at 24 h after training in order to evaluate fear memory consolidation but without receiving footshocks. The bifactorial ANOVA analyze a possible interactive effect between stress and/or fear learning (PSTD model) in delta chemokine levels; for this purpose, “stress factor” and/or “fear learning (CFC)” effect/s were evaluated by including four groups (UND = control, ST, CFC24, ST + CFC24).

**Figure 1:**
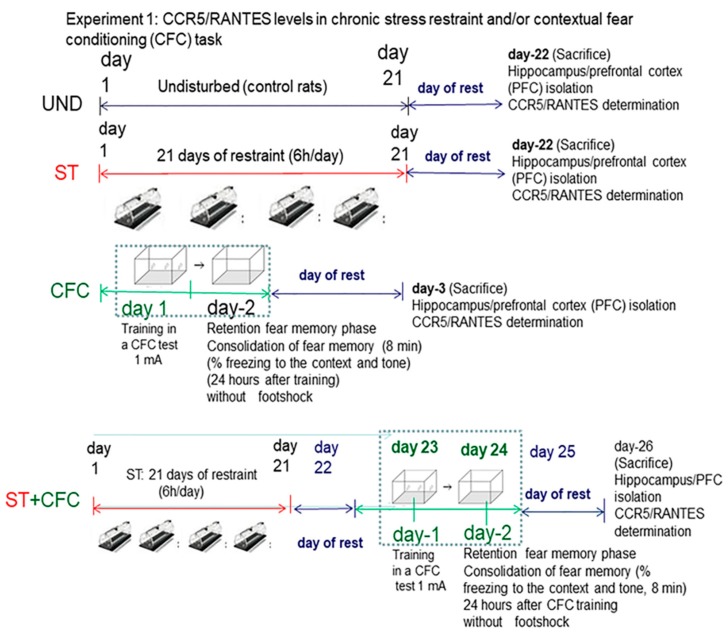
Behavioral protocol for chronic stress and/or contextual fear conditioning.

These CCR5/RANTES chemokines were measured by ELISA (hippocampus/prefrontal cortex) and Western blot in crude synaptosomes. The experimental design included these groups: (i) control (UND: undisturbed rats, *n* = 8) that did not receive treatment/s, (ii) animals subjected to 21 days of chronic stress restraint in plastic restrainers (ST: 6-h/day, *n* = 8); (iii) contextual fear conditioning (CFC)-trained rats received the footshocks (1 mA); chemokines were measured by ELISA (crude synaptosomes from hippocampus/PFFC) in rats re-exposed to the context at 24 h post-testing (CFC24, *n* = 8). The levels of freezing response to the context and tone were evaluated as an index of fear memory consolidation at 24 h after the CFC session (*n* = 8). (iv) Rats subjected to 21 days of chronic stress restraint (6-h/day) and subsequently, 1 day after the last restraint session, these animals were CFC trained with 1 mA of footshocks (ST + CFC24, *n* = 8); rats were re-exposed to the training context at 24 h after the CFC session (CFC24) without receiving footshocks, following own protocols [28]. After the last stress session, the rats were returned to their respective cages. The undisturbed rats (UND: controls) remained in their home cages without behavioral manipulation (see design in Figure 1). All rats were sacrificed 24 h after their last experimental condition. Undisturbed controls (UND) were sacrificed at the same time as the rest of the groups. Chemokines were evaluated by ELISA in crude synaptosomes (hippocampus/prefrontal cortex) at 24 h after the CFC session.

Since structural alterations have been demonstrated in the hippocampus [12] and prefrontal cortex of rats subjected to 21 days of chronic stress restraint [6], we evaluated whether levels of these chemokines could be affected by chronic stress and/or fear memory consolidation at 24 h after a CFC session.

#### 3.1.1. Hippocampus: Increased Hippocampal CCR5 Levels in Chronically Stressed Rats 24 Hours after Contextual Fear Conditioning Training (24 Hours Post-testing)

**Figure 2:**
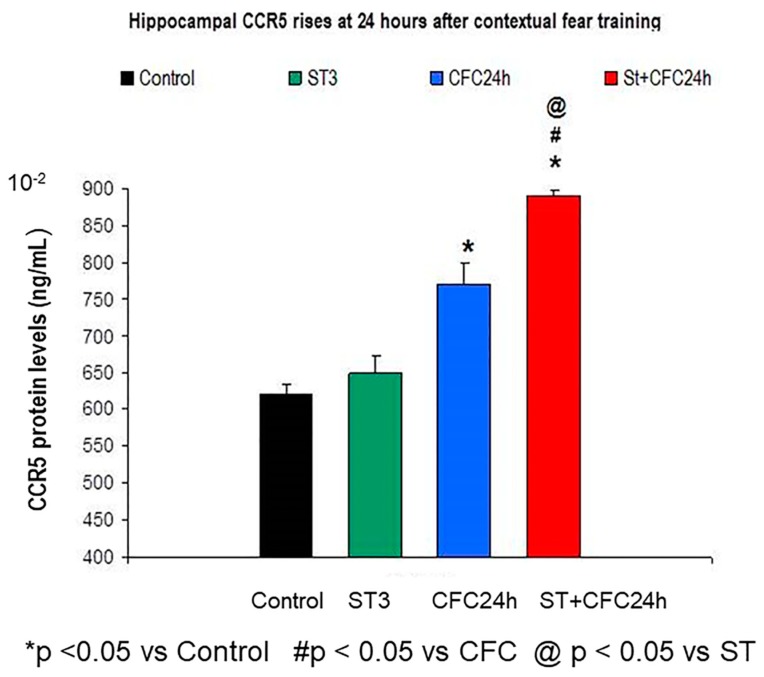
CCR5 protein levels in crude synaptosomes from the hippocampus of chronic stress and/or CFC-trained rats (24 h after the CFC session).

Bifactorial ANOVA revealed higher hippocampal CCR5 levels at 24 h after the CFC training session (F (1, 31) = 0.3 *p* < 0.05) as well as a significant effect for the “stress factor” (F (1, 31) = 0.3; *p* < 0.05); the interaction between the two factors also increased CCR5 protein levels “(F (1, 31) = 0.014; *p* = 0.049); the post hoc Bonferroni also revealed elevated CCR5 in stressed animals that were trained in a CFC paradigm compared to CFC-trained rats (without stress, *p* < 0.05) as well as compared to undisturbed control rats (*p* < 0.05, Figure 2).

#### 3.1.2. RANTES (Hippocampus)

**Figure 3:**
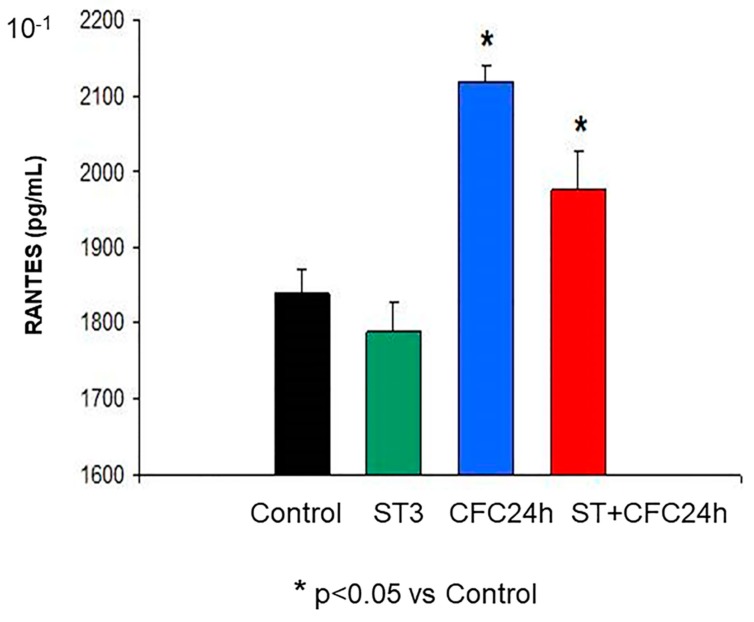
Increased RANTES protein levels 24 after CFC post-testing.

The ANOVA revealed a significant effect for fear memory consolidation (F (1, 31) = 23.74; *p* = 0.000) as well as for the “stress factor” (F (1, 31) = 0.7, *p* < 0.05); however, there was no interactive effect between these factors (F (1, 31) = 8.01; *p* = 0.97; n.s). The Bonferroni post hoc test revealed higher RANTES levels at 24 h post-CFC session than in control animals (without stress, *p* < 0.05, Figure 3). In addition, chronically stressed rats that were CFC-trained had higher RANTES levels than controls (*p* < 0.05).

#### 3.1.3. Increased IL-6 Levels by Fear Learning in the Hippocampus of Chronically Stressed Rats 24 Hours after CFC Training with 1 mA

**Figure 4:**
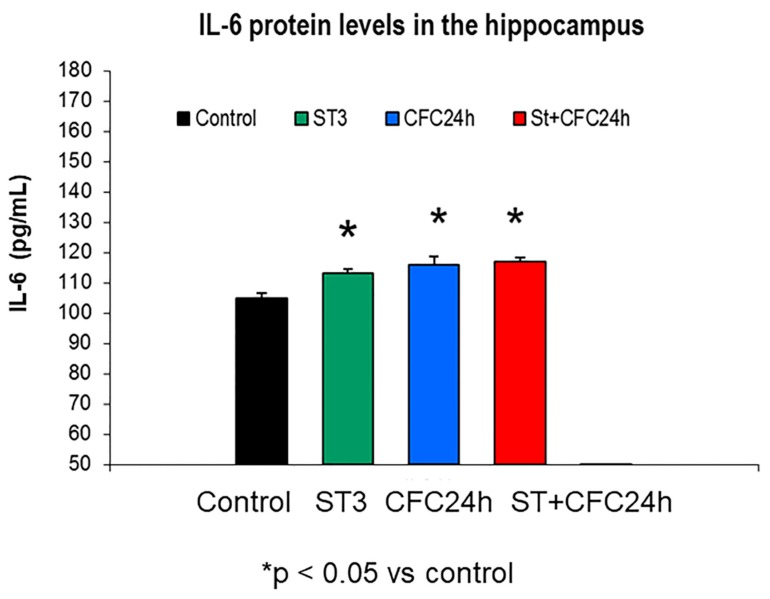
Increased IL-6 levels in the hippocampus of chronically stressed rats as well as higher levels 24 h after the CFC session.

The Mann–Whitney analyses revealed augmented IL-6 protein levels in homogenates from the hippocampus by chronic stress; in fact, IL-6 protein level elevations were observed in chronically stressed rats (*p* < 0.05) as well as 24 h after CFC training as compared to controls (without stress, *p* < 0.05, Figure 4).

The Figure 4 shows hippocampal mean ± S.E.M (standard error of mean) of IL-6 protein levels (Figure 4); The groups are also indicated in CCR5 levels (Figure 2), RANTES levels (Figure 3) in the hippocampus of chronically stressed animals and/or fear memory consolidation at 24 h after the CFC session (*n* = 8 rats/group).

UND: Undisturbed control rats did not receive treatment/s (*n* = 8).

ST: Rats subjected to 21 days of chronic stress restraint in plastic restrainers (6-h/day, *n* = 8).

CFC24: Rats trained in a contextual fear conditioning paradigm CFC (1 mA of footshocks) and re-exposed to the conditioning chamber 24 h after CFC (without receiving footshocks).

ST + CFC24: Rats subjected to 21 days of chronic stress restraint were trained in a contextual fear conditioning paradigm (CFC, 1 mA). These animals were re-exposed to the conditioning chamber 24 h after the CFC session (*n* = 8); these beta chemokines were evaluated by ELISA 24 h after the CFC session in crude synaptosomes (hippocampus).

#### 3.1.4. Prefrontal Cortex: Chronic Stress and/or Contextual Fear Conditioning (CFC) did not Affect CCR5/RANTES Protein Levels in the Prefrontal Cortex 24 h after the CFC Session

**Figure 5:**
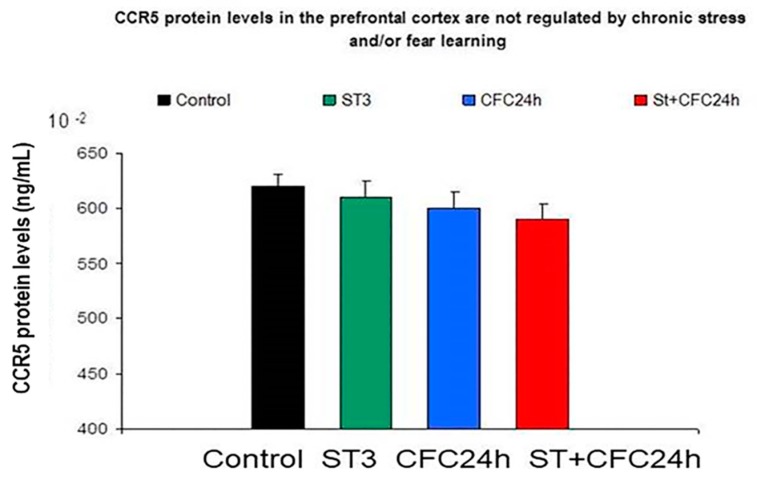
CCR5 protein levels in the prefrontal cortex are not regulated by chronic stress restraint or fear learning in rats.

With regard to CCR5 levels in the prefrontal cortex, the ANOVA did not shown an effect for “stress factor” (F (1, 31) = 0.3; *p* = 0.58; n.s) and there was a lack of effect from the “conditioning factor” (F (1, 31) = 0.18, *p* = 0.66; n.s). We found a slight tendency for interaction between both factors F (1, 31) = 2.565, *p* = 0.115; n.s). Similar results were observed for RANTES levels in a bifactorial ANOVA in the PFC (data not shown). The post hoc Tukey test showed a tendency toward lower levels in the cortex of chronically stressed rats 24 h after the CFC session as compared to chronically stressed rats (*p* = 0.1; n.s; Figure 5).

Figure 5 shows no effect for CCR5 protein levels in the prefrontal cortex of rats subjected to chronic stress restraint as well as a lack of effect for fear learning. CCR5 was evaluated in crude synaptosomes (PFC, *n* = 8 animals/group). The graph indicates mean values ± S.E.M.

UND: Undisturbed (control rats) did not receive treatment/s (*n* = 8).

ST: Rats subjected to 21 days of chronic stress restraint in plastic restrainers (6-h/day, *n* = 8).

CFC24: Rats trained in a contextual fear conditioning paradigm CFC (1 mA of footshocks) and re-exposed to the conditioning chamber 24 h after CFC (without receiving footshocks).

ST + CFC24: Rats subjected to 21 days of chronic stress restraint were trained in a contextual fear conditioning paradigm (CFC, 1 mA). These animals were re-exposed to the conditioning chamber 24 h after the CFC session (*n* = 8); these beta chemokines were evaluated by ELISA 24 h after the CFC session in crude synaptosomes (hippocampus/PFC).

### 3.2. Experiment 2: Effect of Fear Learning in RANTES Levels (12 and 24 h Post-testing)

**Figure 6:**
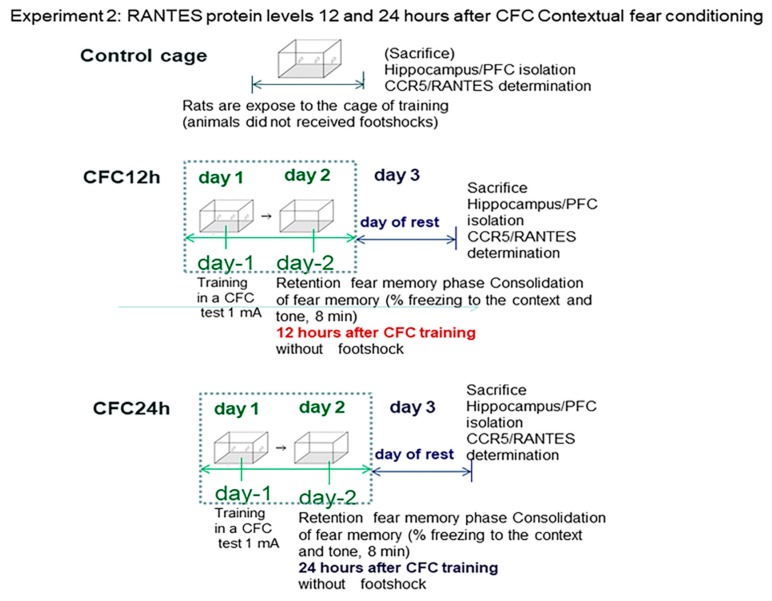
Behavioral protocol for beta chemokine detection in contextual fear learning.

In Experiment 2, rats were not subjected to chronic stress restraint. We evaluate whether RANTES could be differentially regulated at two time windows after the CFC session (12 and 24 post-testing). For this aim, rats were trained with 1 mA of footshocks in a CFC paradigm. Rats trained with 1 mA footshocks were re-exposed to the context at 12 and 24 h after the CFC session (without receiving further footshocks). Fear memory consolidation was measured as an index of fear memory consolidation (12 and 24 h after the CFC session) and RANTES levels were measured at 12 and 24 h after the CFC training session without receiving any further footshocks (n = 7 rats/group). The undisturbed control cage (UND) included rats exposed to the cage conditioning chamber but without receiving footshocks (Figure 6).

#### 3.2.1. Increased Corticosterone and Enhanced Freezing Percentage to the Context at 12 and 24 h after the CFC Session with 1 mA Footshocks

**Figure 7:**
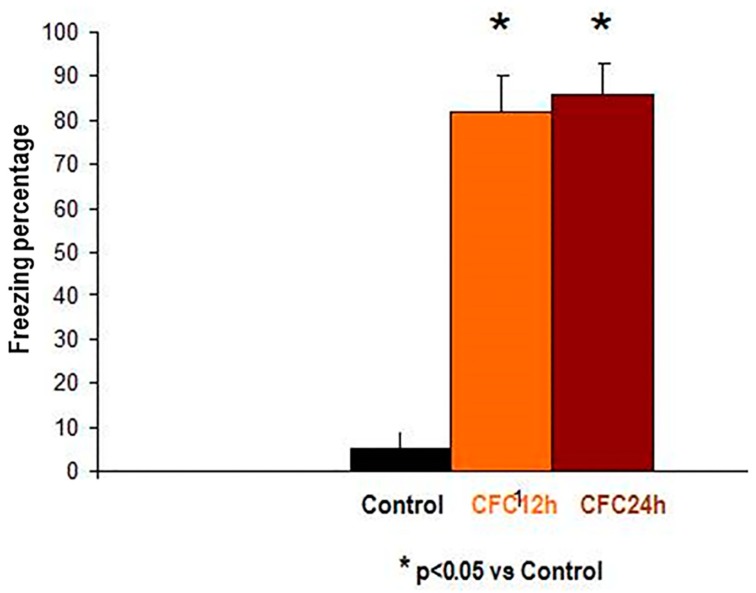
Increased freezing levels at 12 and 24 h after the CFC session. * *p* < 0.05 vs. undisturbed rats (exposed to the cage).

We observed increased plasma corticosterone levels and enhanced freezing percentage to the context at 12 and 24 h after the CFC session (*p* < 0.05, Figure 7) as compared to controls, in agreement with own previous findings [27,28].

Figure 7 shows the mean ± S.E.M freezing percentage to the context at 12 h (orange) as well as 24 h (brown) after the CFC session with 1 mA footshocks (Figure 7). Freezing is an index of fear memory consolidation (*n* = 8 rats/group). Controls (black) did not receive footshocks and are undisturbed controls; the rats were only expose to the cage without receiving electric footshocks. Freezing percentages to the context were measured at 12 and 24 h after CFC training. The results are shown as mean ± S.E.M.

Control: Control rats exposed to the cage conditioning chamber (*n* = 5).

CFC12: Rats trained in a contextual fear conditioning paradigm (1 mA of footshocks) and re-exposed to the conditioning chamber at 12 h (CFC12, *n* = 7) or 24 h (CFC24, *n* = 7) after the CFC session but without receiving footshocks (*n* = 7).

#### 3.2.2. Increased RANTES Protein Levels in the Hippocampus 12 and 24 Hours after CFC Training with 1 mA of Electric Footshocks

**Figure 8:**
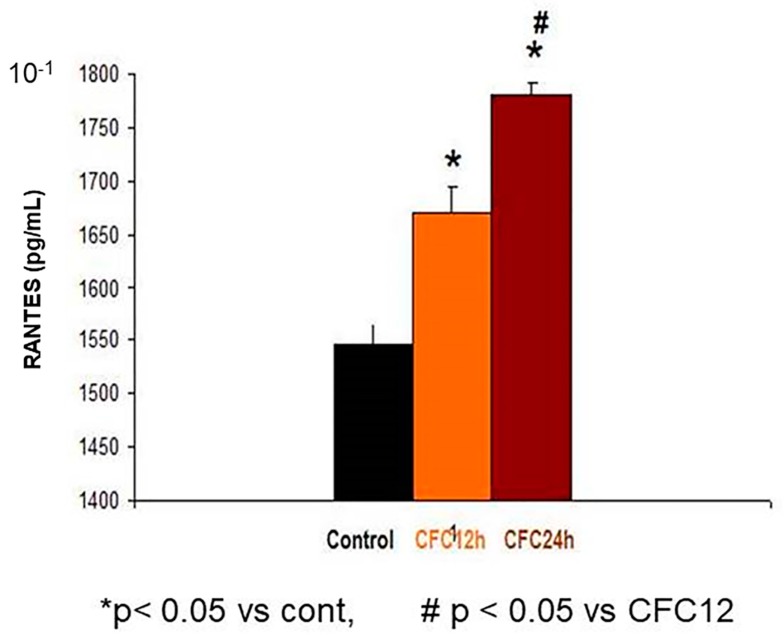
Increased RANTES levels at 12 and 24 h after the CFC session.

The Bonferrroni analysis revealed CCR5/RANTES elevations in the hippocampus at 12 and 24 h after the CFC session as compared with control rats exposed to the cage (without receiving footshocks, *p* < 0.05 in both cases). Interestingly, RANTES levels were significantly higher at 24 h after CFC as compared with CFC at 12 h post-training values (Figure 8).

Control: Control rats exposed to the cage conditioning chamber (*n* = 5) without footshocks.

CFC12: Rats trained in a contextual fear conditioning paradigm (1 mA of footshocks) and re-exposed to the conditioning chamber at 12 h (CFC12, *n* = 7) or 24 h (CFC24, *n* = 7) after the CFC session but without receiving footshocks (*n* = 7).

RANTES levels were measured at 12 and 24 h after the CFC session. Figure 7 shows the mean (RANTES) ± S.E.M values at 12 and 24 h CFC post-testing (*n* = 8 animals/group).

### 3.3. Experiment 3: Effect of CCR5 Blockade by Maraviroc in Corticosterone and Freezing Levels (24 Hours after CFC Training Session)

**Figure 9:**
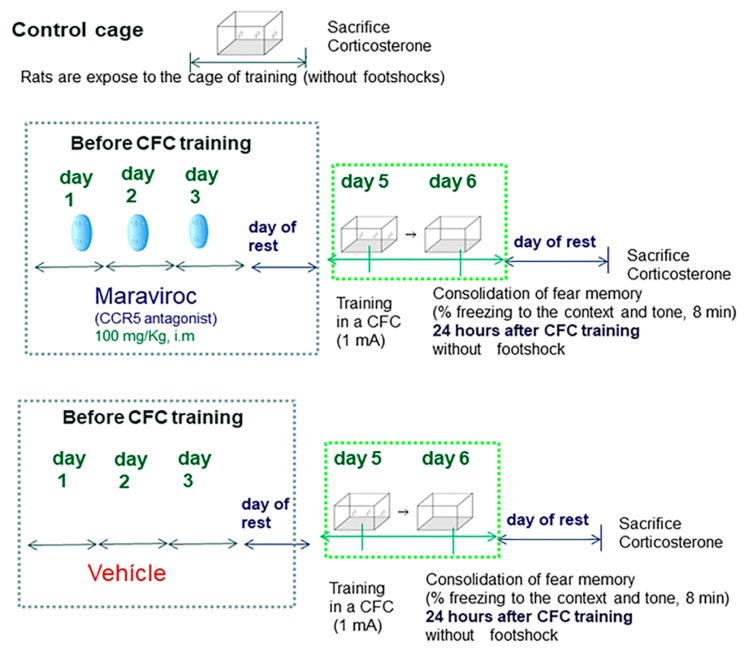
Maraviroc increased freezing percentage to the context and elevated corticosterone levels in CFC-trained rats.

As the RANTES rises seem to be associated with fear memory consolidation at 24 h after the CFC session, we evaluated whether CCR5 blockade by maraviroc affects corticosterone levels and alters freezing levels (context and tone) as compared to vehicle CFC-trained rats. For this purpose, rats received 1 mA of footshocks during CFC training following our own protocols [28]. The maraviroc was i.m administered (*n* = 7, a CCR5 blocker, i.m: 100 mg/Kg) to rats for 3 consecutive days before CFC fear training with 1 mA of footshock intensity. The vehicle-treated rats received vehicle for 3 consecutive days (saline, i.m) before CFC training (*n* = 7). These rats were trained in a CFC with 1 mA of footshock intensity; both experimental groups were re-exposed to the context in the conditioned chamber box 24 h after the CFC session without receiving any further footshocks. Corticosterone and freezing percentage (context and tone) were compared between maraviroc and vehicle CFC-trained rats at 24 h after the CFC session. Freezing percentage (context and tone) were evaluated during 8 min as an index of fear memory consolidation (*n* = 7/group) but these animals did not receive footshocks (Figure 9).

#### 3.3.1. Maraviroc i.m Injections for 3 Consecutive Days before CFC Training (1 mA Footshocks) Increased Corticosterone Levels and Enhanced Freezing to the Context at 24 Hours after the CFC Session as Compared to Vehicle CFC-Treated rats (1 mA)

**Figure 10:**
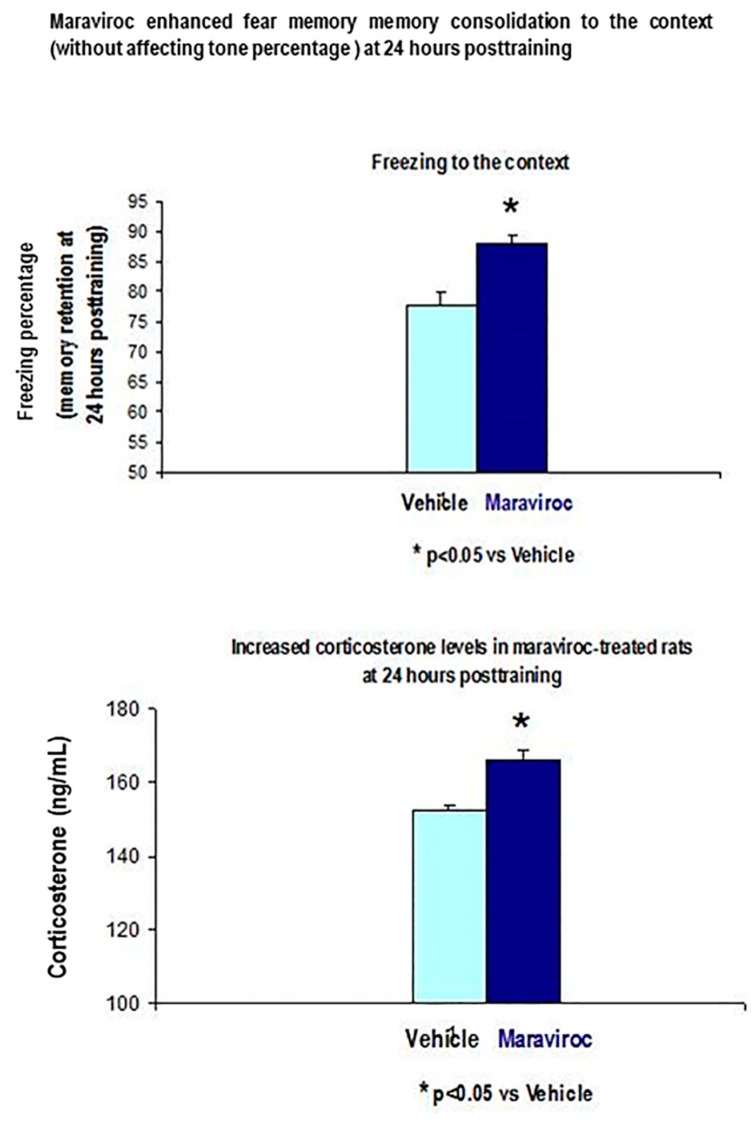
Enhanced freezing to the context in maraviroc-trained rats together increased plasma corticosterone levels, 24 h after CFC training session as compare to vehicle CFC-trained rats with 1 mA footshocks (*p* < 0.05, *n* = 7 animals/group, *p* < 0.05). Maraviroc (3 days, i.m: 100 mg/Kg) treatment before CFC training enhanced freezing percentage to the context when rats were re-exposed to the conditioning chamber at 24 h after the CFC sessions compared to vehicle CFC-trained rats (1 mA of footshocks).

These maraviroc-trained rats (100 mg/Kg, i.m) had increased corticosterone levels as well as enhanced freezing percentage to the context at 24 h after the CFC session as compared to vehicle CFC-trained rats (*p* < 0.05, Figure 10).

Figure 10 showed increased corticosterone levels and higher freezing percentage to the context at 24 h after the CFC session in maraviroc-treated rats.

#### 3.3.2. Maraviroc i.m Injections for 3 Consecutive Days before CFC Training (1 mA Footshocks) did not Prevented RANTES Overexpression as Compared to Vehicle CFC-Treated Rats (1 mA)

**Figure 11:**
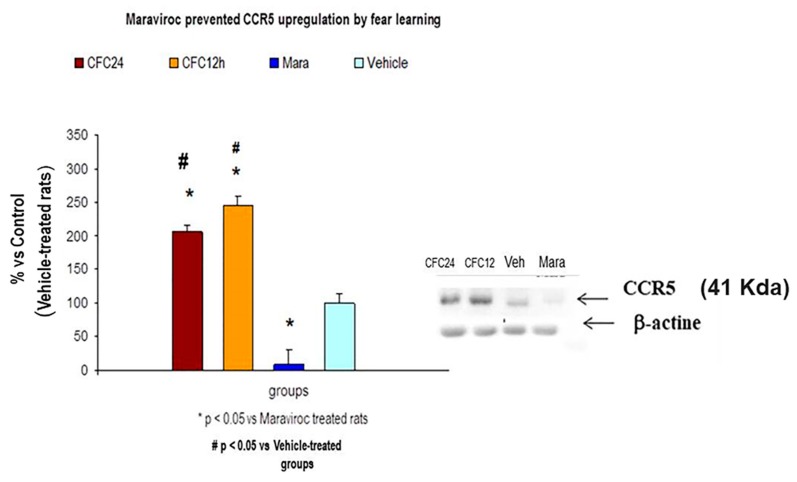
Maraviroc induced an almost complete CCR5 blockade in maraviroc CFC-trained rats as compared to vehicle-treated animals (*n* = 5 rats/group, *p* < 0.05). Vehicle-treated rats are expressed as 100% of band signal (densitometry). All data are expressed as percentage control (vehicle percentage). CCR5 is upregulated by fear memory (12 and 24 h after CFC session) and maraviroc prevented these upregulations (see Western blot (41 Kda band for CCR5) and quantification). Bands for CCR5 were normalized with beta actine. Mara: Maraviroc CFC-treated rats (100 mg/Kg, i.m); Vehicle: Rats received vehicle and these animals were trained in a CFC paradigm (1 mA); CFC12h: Fear memory consolidation at 12 h after CFC session; CFC24h: Fear memory consolidation at 24 h after CFC session.

RANTES levels were still higher in the hippocampus of maraviroc-trained rats, 24 h after CFC training session, as compared to vehicle CFC-trained rats with 1 mA footshocks (*p* < 0.05, *n* = 7 animals/group, *p* < 0.05). The Western blot analysis showed increased CCR5 protein levels 12 and 24 h after CFC session. This 41 Kda band for CCR5 was almost completely abolished by maraviroc treatment although there was a slight signal as compared to vehicle-treated rats. The Western blot indicated all densitometry (*n* = 5/group). CCR5 increased 12 and 24 h after the CFC session and maraviroc prevented this upregulation (*n* = 5–7 rats/group). All data were expressed as a percentage of the vehicle-control group, which is 100% (Figure 11).

#### 3.3.3. Maraviroc i.m Injections for 3 Consecutive Days before CFC Training (1 mA Footshocks) did not Affect IL-6 (24 Hours Post-testing) Protein Levels as Compared to Vehicle-CFC Treated Rats (1 mA)

**Figure 12:**
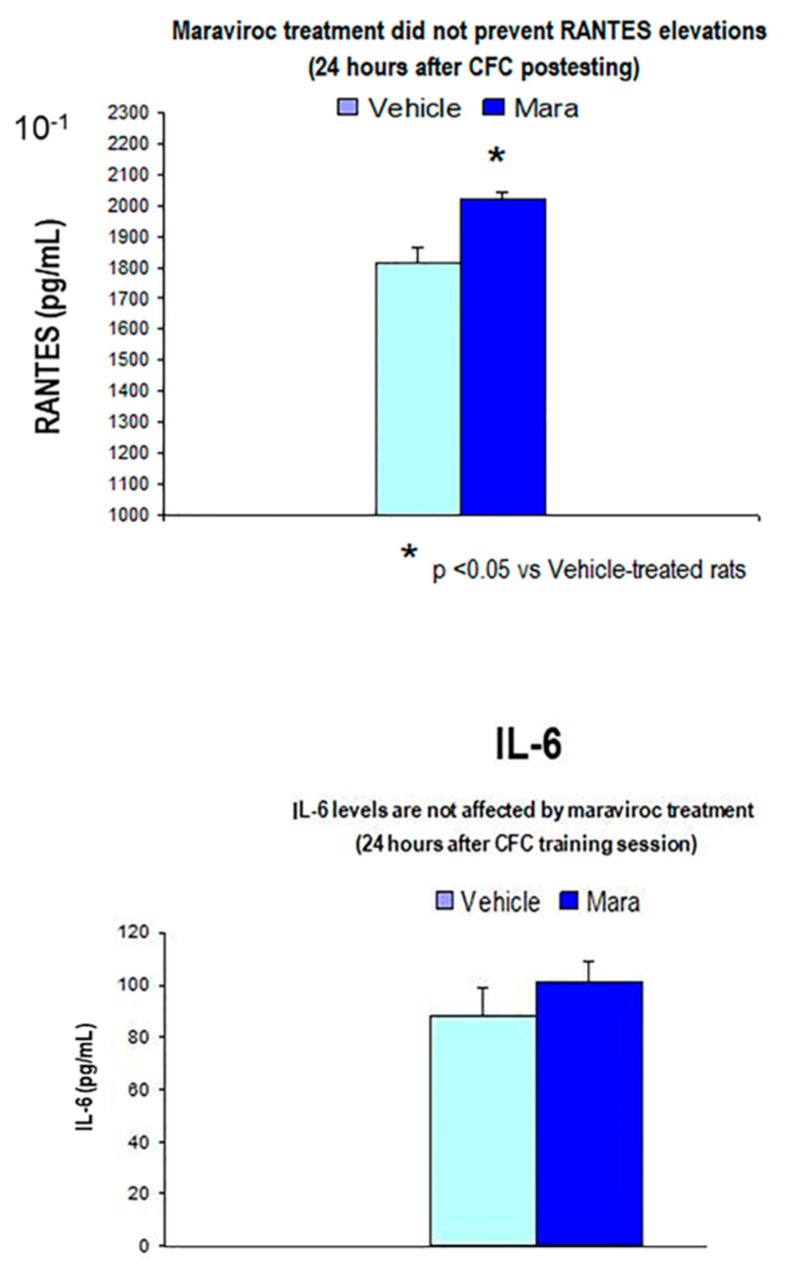
Maraviroc did not prevent RANTES elevations (mean ± S.E.M) in CFC-trained rats as compared to vehicle-treated animals (*n* = 7 rats/group, *p* < 0.05). In addition, IL-6 levels did not differ between both experimental groups (*p* > 0.05. n.s, *n* = 7 rats/group).

Maraviroc treatment augmented RANTES levels at 24 h after the CFC training session although IL-6 levels did not differ in maraviroc-treated rats as compared to vehicle CFC-trained animals (*p* > 0.05, n.s, *n* = 7/group, Figure 12).

In order to evaluate whether the CCR5 blockade affects corticosterone levels under basal conditions (in the absence of stress or learning), we compared this stress hormone in maraviroc (100 mg/Kg, i.m., *n* = 5)-treated controls and unstressed controls (without CFC training, *n* = 5). Our results indicated that corticosterone levels did not differ between both groups (145 ± 18 (control undisturbed) vs. 156 ± 19 (maraviroc) ng/mL, *p* > 0.05, n.s, Figure 12).

## 4. Discussion

The specificity of RANTES elevations in the rat hippocampus but not in the prefrontal cortex could contribute to fear memory consolidation at 12 and 24 h after a CFC session; however, hippocampal/prefrontal CCR5/RANTES levels did not differ between rats subjected to chronic stress restraint and controls (without stress). The Pavlovian principle associates an innocuous cue (context or tone) with an aversive stimulus (electrical footshock). When rats were re-exposed 24 h later to the context (without electrical footshocks), the IL-6 levels were higher in the hippocampus of chronically stressed rats, suggesting that CFC training contributed to increased IL-6 levels (ST + CFC24) in chronically stressed rats as compared to non-footshocked stressed rats. These hippocampal Il-6 elevations in footshock-trained rats could reflect Pavlovian conditioning (fear memory consolidation) at 24 h after the CFC training session [30]. This elevations in proinflammatory cytokine, like Il-6 level [31], contribute to aversive conditioned behavior; these findings in fear learning are in concordance with the increased production of Il-6 by peripheral blood mononuclear cells observed in PSTD patients [30]. In addition, exposure to psychological stressors elevates plasma IL-6 levels [31]; although CCR5 glial chemokine activation after axonal injury associated with entorhinal cortex lesions directs leukocytes [32], these beta chemokines (hippocampal/cortex) levels did not differ between stressed and control rats.

Chemokines regulate neuronal migration [33] and are also play a role in neuroinflammation and neurodegeneration [34,35]. CCR5/RANTES chemokines were measured in crude synaptosomes since chemokine ligands (like SDF 1 alpha) can be rapidly secreted by neurons and transported to synapses after their synthesis [23]. The CCR5/RANTES elevations could induce neuroplastic changes involved in fear memory consolidation at 24 h after the CFC session. ELISA data showed increased CCR5 protein levels after the CFC session (12 and 24 h post-testing); maraviroc treatment before the CFC learning abolished this upregulation. Since certain chemokines, i.e., stromal derivate factor 1 (CXCL12 = SDF1 Alpha, the ligand for CXCR4) can guide neuronal migration by interacting with specific chemokine receptors in the hippocampus [36,37] and prefrontal cortex [38], we suspect that these hippocampal elevations contribute to fear learning consolidation. In fact, the addition of RANTES recombinant to an NT-2 cell line in vitro induced synaptogenesis and neurite outgrowth [39]. In addition, chemokine SDF-1 = (CXCL12) differentially regulates axonal elongation and branching in hippocampal neurons [40]. The CCR5 rise enhanced long-term memory consolidation in the hippocampus of trained rats in passive avoidance (another aversive learning paradigm) [41]. These data agree with CCR5 elevations in the hippocampus of CFC-trained rats 24 h after the CFC session. Chemokines contribute neural-activity-dependent changes and PSA-NCAM (polysialylated-cell adhesion molecule) also contributes to traumatic memory consolidation in rats [26,28,29,42]. As dendrite-selective redistribution of chemokine receptors (i.e, CXCR4) has been observed following agonist stimulation [43], these RANTES elevations could promote neuroplastic changes via CCR5 during traumatic memory (fear memory) consolidation. Maraviroc (a CCR5 antagonist) treatment before fear learning increased corticosterone levels and also enhanced fear memory consolidation (to the context) at 24 h after the CFC session as compared to vehicle CFC-trained rats. The findings concur with the enhanced freezing responses to the context reported in CFC-trained mice lacking CX3CR1 (transgenic mice lacking this delta chemokine receptor) [44]. Moreover, the enhanced fear memory in maraviroc CFC-trained rats agrees with the improved cognitive responses of viral-suppressed chronic HIV seropositive patients (suppressed viral load) following CCR5 antagonism by maraviroc [43]. Maraviroc crosses the blood–brain barrier and it specifically blocks CCR5 but not CCR1 [45]. Thus, enhancement of freezing responses at 24 h after the CFC session produced by maraviroc do not exclude the possibility that RANTES could bind to CCR1 in the hippocampus. As RANTES levels were still higher in maraviroc-treated rats as compared to vehicle-treated animals, we should not exclude that RANTES levels could have provoked desensitization on other chemokine receptors (i.e, CCR1). For example, Met-RANTES (a CCR5 antagonist) internalized CCR5 in a slower, less potent manner than the agonists CCL3 and RANTES **=** CCL5, [46]. Met-RANTES (a CCR5 antagonist) is also capable of partial agonist activity regarding receptor signaling and internalization. As receptor trafficking impacts on cell surface expression and the ability of the receptor to respond to more ligands, this information may indicate an alternative regulation of CCR5 by Met-RANTES that allows the modified ligand to reduce inflammation through stimulation of a pro-inflammatory receptor. In addition, chemokine receptor deficiency is associated with increased levels of ligands (fractalkine **=** CX3CL1) in circulation and tissues in transgenic lacking CX3CR1 -/- [47]. In our study, Il-6 levels did not differ between maraviroc CFC-trained rats and vehicle-treated animals. There is controversy about the involvement of CX3CR1 (a delta chemokine receptor) in contextual fear learning; two studies have analyzed the role of CX3CR1 in the hippocampus of transgenic mice lacking CX3CR1. Schubert I et al. (2019) reported increased post-shock freezing in CX3CR1-/- transgenic mice, which expressed significantly higher post-reminder shock freezing as compared with CX3CR1+/+ wild type mice [8]. These findings are in concordance with higher freezing responses (to the context) found in maraviroc-treated rats when CCR5 was blocked. In fact, when rats were exposed to the context at 24 h after CFC training, stronger fear memory consolidation and higher RANTES and corticosterone levels were observed in maraviroc CFC-trained rats than in vehicle CFC-trained animals. The indirect evidence leads to the suspicion that CCR5 blockade enhances fear memory consolidation, which concurs with Schubert’s findings in CX3CR1 transgenic mice [8]. Conversely, our findings are in contraposition to Rogers and coworkers’ evidences [48]. The latter reported reduced freezing to the context in transgenic mice lacking CX3CR1 as compared to wild-type mice by CFC fear learning [48]. When exposed to the shock context at 24 h post-training, CX3CR1 transgenic mice showed defective synaptic plasticity and reduced fear memory consolidation [48]. Moreover, CX3CR1-deficient mice have impaired long-term potentiation, an electrophysiological model of learning and memory [48]. The indirect evidence suggest that CCR5 blockade increases fear memory consolidation as well as corticosterone levels. In fact, RANTES levels are still higher in maraviroc-treated rats as compared to vehicle CFC-trained rats. This CCR5 blockade by maraviroc did not affect Il-6 proinflammatory cytokine levels.

Neurobiological studies support the altered function of limbic brain areas regulating stress and emotional responses, such as the hippocampus and the amygdala, in PTSD [49]. PTSD patients have impaired processing of fear, leading to re-experiencing, avoidance, and symptoms of hyperarousal to trauma reminders. The inability to extinguish intense fear memories is a clinical problem in patients with psychiatric disorders involving dysregulation of fear, phobias, and panic disorder [49,50,51]. Cognitive therapy and psychostimulants (nootropic, stabilizing mood drugs, or antipsychotics) could prevent anxiety-related disorders in PSTD [50]. The CCR5/RANTES axis emerges as a possible pharmacological target in neuropsychiatry.

## 5. Conclusions

The CCR5/RANTES chemokine elevations in the hippocampus, 12 and 24 h after CFC training, could contribute to fear memory consolidation. When CCR5 was blocked by maraviroc before CFC training, plasma corticosterone levels and fear memory consolidation were greater than in vehicle CFC-trained rats at 24 h after the CFC session. In addition, RANTES levels were still higher by CCR5 blockade in maraviroc CFC-trained rats as compared to vehicle CFC-trained animals. Conversely, IL-6 levels were not affected by maraviroc treatment.
